# De novo transcriptome sequencing and analysis of *Anisakis pegreffii* (Nematoda: Anisakidae) third-stage and fourth stage larvae

**DOI:** 10.21307/jofnem-2020-041

**Published:** 2020-04-16

**Authors:** U-Hwa Nam, Jong-Oh Kim, Jeong-Ho Kim

**Affiliations:** 1 Department of Marine Bioscience, College of Life Science, Gangneung-Wonju National University, Gangneung, 25457, Korea; 2 Institute of Marine Biotechnology, Pukyong National University, Busan, 48513, Korea

**Keywords:** *Anisakis pegreffii*, transcriptome, next generation sequencing, differentially expressed genes, genomics

## Abstract

*Anisakis pegreffii* is known as one of the causes of a fish-borne zoonosis, anisakidosis. Despite its significant public health and food hygiene impacts, little is known of the pathogenesis, genetic background of this parasite, at least partly due to the lack of genome and transcriptome information. In this study, RNA-seq and de novo assembly were conducted to obtain transcriptome profiles of the *A. pegreffii* third and fourth larvae. The third stage larvae (APL3) were collected from chub mackerel and the fourth stage larvae (APL4) were obtained by in vitro culture. In total, 47,243 and 43,660 unigenes were expressed in APL3 and APL4 transcriptomes. Of them, 18,753 were known and 28,490 were novel for APL3, while 18,996 were known and 24,664 were novel for APL4. The most abundantly expressed genes in APL3 were mitochondrial enzymes (COI, COII, COIII) and polyubiquitins (UBB, UBIQP_XENLA). Collagen-related genes (col-145, col-34, col-138, Bm1_54705, col-40) were the most abundantly expressed in APL4. Mitochondrial enzyme genes (COIII, COI) were also highly expressed in APL4. Among the transcripts, 614 were up-regulated in APL3, while 1,309 were up-regulated in APL4. Several protease and protein biosynthesis-related genes were highly expressed in APL3, all of which are thought to be crucial for invading host tissues. Collagen synthesis-related genes were highly expressed in APL4, reflecting active biosynthesis of collagens occurs during moulting process of APL4. Of these differentially expressed genes, several genes (SI, nas-13, EF-TSMT, SFXN2, dhs-27) were validated to highly transcribed in APL3, while other genes (col-40, F09E10.7, pept-1, col-34, VIT) in APL4. The biological roles of these genes in vivo will be deciphered when the reference genome sequences are available, together with in vitro experiments.

The family Anisakidae comprises the nematode species whose adult stages can be found in aquatic animals, while the third stage larvae (L3) generally exist in the body cavity, visceral organs and muscles of various fish and squid species. Although humans are not the final hosts of these nematodes, the larval forms of anisakid nematodes, particularly those of the genera *Anisakis* and *Pseudoterranova* are known to be associated with human infection by the ingestion of raw or undercooked fish or cephalopods harboring these larvae (Mattiucci and D’Amelio, 2014). Infection with these nematodes is therefore considered to be a threat to public health due to their zoonotic potential and the presence of larvae in fish products cause aesthetic problems, reducing commercial value. Many studies have also revealed that alive or dead anisakid larvae can cause allergic reactions to humans, which are often associated with elevated level of immunoglobulins E (Audicana and Kennedy, 2008; Nieuwenhuizen and Lopata, 2013).

Of genus *Anisakis*, *Anisakis simplex* sensu lato (s.l.) has been traditionally considered as the main causative agent of anisakidosis (Audicana and Kennedy, 2008). But since molecular approaches have been introduced for identification of anisakid nematodes, it was proved that there are three species in *A. simplex* complex and of them, the two sibling species *A. simplex* sensu stricto (s.s.) and *A. pegreffii* were shown to be the causative agent of human infection (Mattiucci and D’Amelio, 2014 and the references therein). Several in vitro and in vivo studies have also demonstrated that *A. pegreffii* has pathogenic potentials to humans, as well as *A. simplex* (s.s.) (Suzuki et al., 2010; Jeon and Kim, 2015). Therefore, more emphasis should be placed on studying epidemiology and pathogenicity of *A. pegreffii* as well.

*Anisakis pegreffii* is known to be widely distributed in the Austral Region between 30°N and 55°S, particularly in the Mediterranean Sea and the East Asia (Umehara et al., 2006; Mattiucci and Nascettii, 2008; Suzuki et al., 2010; Bak et al., 2014; Jeon et al., 2016). Many interemediate/paratenic hosts and definitive hosts of *A. pegreffii* are known to be shared by *A. simplex* (s.s.), and recombinant genotypes of these two sibling *Anisakis* species are reported to sympatrically exist in the waters mentioned above (Abollo et al., 2003; Umehara et al., 2006; Farjallah et al., 2008; Cavallero et al., 2014). In several human clinical cases, *Anisakis* larvae removed from patients were identified as *A. pegreffii* by molecular methods, reflecting their pathogenic potentials to humans (Mattiucci et al., 2013).

Recent impressive progress in genome-wide analyses of socio-economically important nematode parasites helped us to better understand the genetic information of them covering the general biology, host-parasite interaction, pathogenicity and development of drug or vaccine candidates. Moreover, the increase of sequencing data have opened a new era in comparative studies in parasitic nematodes, thereby offering many useful genetic information from different species or closely related species with which to compare pathogenesis, conduct differential diagnosis and a large scale epidemiology (Cantacessi et al., 2015, Lv et al., 2015). Currently, 134 parasitic or free-living nematodes genomes or transcriptomes are available in databases (http://www.wormbase.com, accessed on November 25, 2019) and the numbers will be increased. For anisakid nematodes, several transcriptomic analyses have been focused on *A. simplex*, a well-known cause of human anisakidosis or on comparative studies with a sibling species, *A. pegreffii* (Baird et al., 2016; Cavallero et al., 2018; Llorens et al., 2018), but not on *A. pegreffii* itself. In this study, we obtained transcriptomes of *A. pegreffii* L3 (APL3) and in vitro*-*induced L4 by RNA-seq and de novo assembly. Then, we compared transcriptomes of these two different stages of *A. pegreffii* to provide detailed information on differences regarding their biology and pathogenicity against their hosts.

## Materials and methods

### Sample preparation

Alive APL3 were obtained from chub mackerel (*Scomber japonicus*), which is known to be the one of the main intermediate/paratenic hosts for *A. pegreffii* in Korea (Bak et al., 2014). Freshly caught chub mackerel were purchased from a local fish market located in the east coast of Korea and immediately transported to the laboratory. The body cavity was longitudinally cut and the viscera were carefully examined for collecting the nematodes.

The collected nematodes were placed in a Petri dish filled with sterile PBS and washed several times to remove any tissue debris. Then, they were observed under a stereomicroscope and actively moving nematodes without any injury were selected for in vitro culture to obtain *A. pegreffii* fourth stage larvae (APL4). Each larva was treated with antibiotic-antifungal solution as described elsewhere before starting in vitro culture (Iglesias et al., 2001). Then, individual larvae were placed in a sterile 24 well tissue culture plate (SPL, Korea) with 1 ml of culture medium (pH 4.0) in each well, at 37˚C with 5% CO_2_ atmosphere. The composition of culture medium and culture conditions were followed as described by Iglesias et al. (2001). The culture medium was changed twice a week and the larvae were checked every day. Each individual L3 were considered to be molted to L4 when the sheath were found by stereoscope, and APL4 is known to be induced within approximately five days in these conditions (Jeon and Kim, 2015).

Each individual APL3 and APL4 were washed with sterile PBS, then individually placed in a 1.5 ml Eppendorf tube and stored at −80˚C until use. Each larva was identified by PCR-RFLP and subsequent sequencing for the mitochondrial cox2 gene according to the method described elsewhere (D’Amelio et al., 2000; Nadler and Hudspeth, 2000). The caudal end of each larva was cut and used for molecular identification, and the rest of them were used for next generation sequencing. The larvae identified as *A. pegreffii* were used for further analysis.

### Library construction and next generation sequencing

Total RNA (1 μg per each sample set) was prepared by homogenizing approximately 50 larvae per each developmental stage with Trizol, following the manufacturer’s protocol (Invitrogen, USA). Prior to mRNA isolation, total RNA samples were treated with DNAse. And the RNA yield and integrity was measured by NanoDrop 1000 (Thermo Scientific, Wilmington, USA) and BioAnalyzer 2100 (Agilent Technology, Santa Clara, USA), respectively.

The TruSeq stranded mRNA sample preparation kit (RS-122-2101, Illumina, San Diego, USA) was used for preparing mRNA sequencing libraries. Poly-A-containing mRNA was purified from 1 μg of total RNA, by using Oligo dT attached magnetic beads. Then the purified mRNA was disrupted into short fragments, and first-stranded cDNAs were synthesized using SuperScript∏— reverse transcriptase (Invitrogen, USA) and random hexamers. cDNA with adaptors ligated to both ends were enriched by PCR. The cDNA library size and quality were electrophoretically evaluated by using the Agilent DNA 1000 kit on a BioAnalyzer 2100. The libraries were subsequently sequenced on an Illumina HiSeq 2500. Image analysis was performed using the HiSeq control software version 2.2.58. Raw data were processed and base calling was performed using the standard Illumina pipeline (CASAVA version 1.8.2 and RTA version 1.18.64).

### De novo assembly and functional annotation

The complete sequences of two sample sets were subjected to the adaptor and quality trimming by FastQC method. The contigs were obtained by the assembly of preprocessed sequences assembly using Trinity (Haas et al., 2013). Then, they were clustered using the CD-HIT and TGIGL, to obtain the unique genes as a reference transcriptome (Pertea et al., 2003; Li and Godzik, 2006). The preprocessed reads were further mapped to the reference transcriptome using BWA and the expression patterns (i.e. FPKM) of each genes/transcripts were obtained using RSEM method (Li and Durbin, 2010; Li and Dewey, 2011). Normalization of the FPKM values was conducted by edgeR using TCC R package (Sun et al., 2013). The reference transcriptomes were annotated by mapping against to NR database using BLASTx and SwissProt/UniProt database using InterProScan. The protein coding regions were also annotated with TransDecoder (Haas et al., 2013). The complete annotations were merged together to improve the annotations of each genes/transcripts using in-house scripting.

### Orthologous cluster analysis

In total, six genomes of nematodes were selected for whole-genome orthologous cluster analysis, i.e. *Ancylostoma ceylanicum*, *Brugia malayi*, *Trichinella spiralis*, *Caenorhabditis elegans*, *A. simplex* along with *A. pegreffii* in our study. Complete proteins sequences of these five nematodes were downloaded from WormBase (http://www.wormbase.org) for those selected nematode species and the orthologous clusters were analyzed using OrthoVenn2 with default options (Xu et al., 2019).

### Differentially expressed genes (DEGs) analyses and GO enrichment analysis of DEGs

For DEG analyses, complete FPKM and edgeR scores were taken, and the statistical significance (*P* value and *Q* value) and fold changes (FC) were calculated, based on the normalized values. The filter cut-offs (i.e., *P* ≤ 0.05, *Q* ≤ 0.05 and FC ≥ 2) were used to select the DEGs/transcripts from the given pairs. Gene Ontology (GO) terms for the identified genes from DEG analysis were determined using the GOA database (Barrell et al., 2009).

### Real-time PCR validation

DEGs in either APL3 or APL4 were validated by real-time PCR analysis. cDNA targeting highly ranked DEGs in each ASL3 and ASL4 transcriptomes were synthesized by using PrimeScript™ RT reagent Kit with gDNA Eraser(Perfect Real Time; Takara, Japan), according to the manufacturer’s instructions. As a reference gene, GAPDH (Glyceraldehyde-3-phosphate dehydrogenase) gene was selected. Total RNA was extracted from each APL3 and APL4 samples as mentioned above; 1 μl of total RNA extracted using Trizol (Sigma. USA) was mixed with 2 μl of 5 X gDNA Eraser Buffer, 1 μl of gDNA Eraser, 6 μl of RNase-free dH_2_O, and incubated at 42˚C for 2 min. Then, cDNA was synthesized by adding 4 μl of 5 X PrimeScript™ Buffer 2, 1 μl of PrimeScript™ RT Enzyme Mix I, 1 μl of RT Primer Mix, 4 μl of RNase-free dH_2_O, and incubated at 37˚C for 15 min.

The selected *A. pegreffii* gene sequences and the internal control primers were retrieved from Genbank and gene-specific primers were designed using Primer Expression 3.0 software (Applied Biosystems, USA). The sequences of the primers used in this study are listed in Table 1. The real-time PCRs were set up using SYBR Premix Ex TaqII (Tli RNase H Plus, Takara, Japan) on 96 well plates in 20 μl reaction volumes consisting 2 ul of 10-fold diluted cDNA as templates, 1.6 μl of primers (10 μM), 0.4μof 50 X ROX reference dye II and 6.0 μl of dH_2_O. The reaction was conducted with an Applied Biosystems 7500 Real Time PCR System (Applied Biosystems, USA) and cycling conditions involved 15 min of initial pre-denaturation step at 95˚C, then 45 cycles as follows: 15 sec at 95˚C, 15 sec 60˚C, and 30 sec at 72˚C. GAPDH gene was used as internal control and each gene was run in triplicates. Relative expression was calculated using the ΔΔCT method (Livak and Schmittgen, 2001) and significant differences were identified with the Student’s *t*-test at *P* < 0.01.

**Table 1.** Primer information for real-time PCR analysis.

Stage	Gene	Primer sequences (5´ − 3´)	Length (bp)
APL3	Acin1	F: 5´-TGAATGACGAAGAAGGGAGATGA-3´	108
		R: 5´-CAGTGTCAAATGAGAATAGCGTTTC-3´	
	SI	F: 5´-GAGGCGATTGCTGGAAACAT-3´	111
		R: 5´-CTTCGTTGGTTCTTTTTGTCGTT-3´	
	lact-2	F: 5´-CACCCCACCATCCTCTCCTT-3´	114
		R: 5´-CCGAGTGTATCTGCGAGGAAA-3´	
	sptl-2	F: 5´-GGCAACCAAGAACGAGTGATG-3´	108
		R: 5´-TCAGCATGGGATTTGCAACA-3´	
	Macf1	F: 5´-ATGCTTGCTCCAGTTCTCCTAAA-3´	125
		R: 5´-ATGGACAGGACGCTCTTCTTG-3´	
	ergic2	F: 5´-CTGTTGAGTTGATGGCTGGAAA-3´	106
		R: 5´-ATTTTGGCACCACTGCTTTTATTAG-3´	
	mgat4b	F: 5´-AATGCCAGCGATTGAAGAGTTT-3´	115
		R: 5´-CGCTGGTGTACGCAAATCATA-3´	
	B036.11	F: 5´-TAATGATTGCTGAATGCGTCTGT-3´	120
		R: 5´-ACACCGAAAGATAACAACGAATACG-3´	
	nas-13	F: 5´-AGCAATAGCAGCAGCGATGA-3´	131
		R: 5´-GTAGCCAATGCTTTT-3´	
	EF-TSMT	F: 5´-TTCTTCTACTCCTCATCGCATCTG-3´	100
		R: 5´-TTATGCTCCTCCAGTGCTTTTG-3´	
	SFXN2	F: 5´-TTAGAATGGCGTTGAAGCAGTAGTAG-3´	130
		R: 5´-AGTATCGGTTCTGACCAGTTTTTTG-3´	
	hyd	F: 5´-CCATCCAGTGAAGAAGGATTCC-3´	116
		R: 5´-GAATAGAGCGGTAAGTAGAGCCTTGA-3´	
	dhs-27	F: 5´-ACGATGAACGATTCGGGTAAGT-3´	140
		R: 5´-GCGTTATCCCCACATTTTCAA-3´	
APL4	vps-11	F: 5´-CTCAACACACCACAATACTCATCAAT	105
		R: 5´-CAGCAACATCAACATCACAACTCA	
	EGF1	F: 5´-CACATCAGCCCAGAAACATACG	106
		R: 5´-ATTTAGACCGTCGCCAGGAA	
	CLINT1	F: 5´-AACGCAAGAATAGGCAAACACA-3´	113
		R: 5´-AGGAGCATCAACAGAAAACAATGAG-3´	
	let-805	F: 5´-CACCGAGCACCGCTATCAA-3´	120
		R: 5´-GAAGGAGGCATCAAGGAAAGTG-3´	
	VIT	F: 5´-AGGCTTATCATTACTTCGTTCACTCA-3´	105
		R: 5´-GTTTGGTTGTCGGTTCAGGTGTA-3´	
	F09E10.7	F: 5´-CCACGCTCAAAAGCCCATT-3´	116
		R: 5´-GGACCAGCGGAAGTTCAGAA-3´	
	ATP8B4	F: 5´-CGACACATTCTTCGTCAGCATT-3´	135
		R: 5´-CCGAACCCAAGCATGAAAA-3´	
	pept-1	F: 5´-TGCTCAAGGAGAAGGAAGTTTACA-3´	113
		R: 5´-TACCCCACTTCACCGATTCG-3´	
	col-34	F: 5´-GCCGAGTAAGCCACAAAACG-3´	121
		R: 5´-TACACCCCGTCCACCTTCA-3´	
	col-40	F: TTCAGCAGGTGGGCAGTCA	98
		R: ATGGAACTCCGGGTGAGGAT	
	SAM-MT	F: 5´-CCATCAAGGCGGCGTTAC-3´	93
		R: 5´-CAGCATGCCATAGATCCAGTGT-3´	

### Identification of putative allergens

To make a list of putative allergens from *A. pegreffii*, the contigs of APL3 and APL4 transcriptomes were compared with the sequence data retrieved from the AllergenOnline Database (Goodman et al., 2016; http://www.allergenonline.com.; version 19), using BLASTp (e-value cut off: <1e-05, identity matching cut off: >70%). In addition, the gene expression level of putative allergens from APL3 and APL4 were compared by DEG analysis with the same conditions as above.

## Results

### Transcriptome profiles of *A. pegreffii* L3 and L4

In total, 57,831,158 (4,395,168,008 bases) and 61,963,202 (4,709,203,352 bases) raw reads were obtained for APL3 and APL4 by Illumina sequencing. The generated raw reads were deposited in the Sequence Read Archive database of NCBI (accession number: PRJNA602791, PRJNA602795). After cleansing and removing low quality reads (*Q* < 30), 53,790,942 (4,079,160,122 bases, APL3) and 56,726,910 clean reads (4,301,151,997 bases, APL4) were obtained (Table 2). In total, 53,383 unigenes were obtained by de novo assembly, with 22,387 known genes and 30,996 novel genes. The average length of unigenes was 1,091 base pairs (data not shown).

**Table 2.** Preprocessing statistics of *Anisakis* transcriptome.

Name	Raw reads		Clean reads	
Reads	Base pairs	Reads (%)	Base pairs (%)
APL3	57,831,158	4,395,168,008	53,790,942 (93.0%)	4,079,160,122 (92.8%)
APL4	61,963,202	4,709,203,352	56,726,910 (91.5%)	4,301,151,997 (91.3%)

**Notes:** APL3: *Anisakis pegreffii* third stage larvae; ASL4: *Anisakis pegreffii* fourth stage larvae

In total, 47, 243 and 43,660 genes were expressed in APL3 and APL4, respectively (> fpkm 1.0). Of these genes, 28,490 and 24,664 genes were novel for APL3 and APL4 (Table 3). The predicted gene number of *A. pegreffii* and several other representative nematodes were compared.

**Table 3.** Gene expression overview of *A. pegreffii* L3 and L4 transcriptomes.

	Gene (fpkm < 1.0)			
Name	Expressed	Known	Novel	Unexpressed
APL3	47,243	18,753	28,490	3,030
APL4	43,660	18,996	24,664	6,613

### Orthologous cluster analysis

The number of orthologous transcripts of *A. pegreffii* and those of other representative nematodes were compared. In total, 8,344  *A. pegreffii* transcripts were orthologous to any of those of the reference nematode groups and 2,549 were common to all the five references (data not shown). Most of these 2,549 transcripts are thought to encode proteins involved in developmental and common biological process of nematodes. In total, 1,022 transcripts did not match with any sequence of the five reference nematode groups, suggesting they may include those putatively encoding novel gene sequences of *A. pegreffii*.

### Highly expressed genes and DEG analyses of *A. pegreffii* L3 and L4 transcriptomes

The abundance of each gene in APL3 and APL4 transcriptome was calculated; ubiquitin genes (UBB, UBIQP_XENLA) were highly expressed in APL3, while collagen genes (col-34, col-138, col-40) were highly expressed in APL4. Several mitochondrial enzyme genes (COI, COII, COIII) were highly expressed both in APL3 and APL4 (Tables 4 and 5).

**Table 4.** Top 20 most abundant unigenes in *A. pegreffii* L3.

No.	ID	Name	Description
1	TBIU006603	COIII	Cytochrome c oxidase subunit 3
2	TBIU013810	COI	Cytochrome c oxidase subunit 1
3	TBIU015558	UBB	Polyubiquitin-B
4	TBIU017350	ZK970.7	Protein ZK970.7
5	TBIU017923	act-2b	Actin-2
6	TBIU015560	UBB	Polyubiquitin-B
7	TBIU017925	act-2b	Actin-2
8	TBIU008478	COII	Cytochrome c oxidase subunit 2
9	TBIU015561	UBIQP_XENLA	Polyubiquitin
10	TBIU002664	UBIQP_XENLA	Polyubiquitin
11	TBIU022916	HSP70	Heat shock 70 kDa protein
12	TBIU007173	NAP1L4	Nucleosome assembly protein 1-like 4
13	TBIU018580	ART2	Putative uncharacterized protein ART2
14	TBIU001952	R09H10.3	Probable 5-hydroxyisourate hydrolase R09H10.3
15	TBIU003351	PCCB	Propionyl-CoA carboxylase beta chain, mitochondrial
16	TBIU022918	HSP70	Heat shock 70 kDa protein
17	TBIU030005	ASP2_ANISI	Serine protease inhibitor 2
18	TBIU005085	Mmadhc	Methylmalonic aciduria and homocystinuria type D homolog, mitochondrial
19	TBIU032617	CLPH_ONCVO	Calponin homolog OV9M
20	TBIU030096	Mcee	Methylmalonyl-CoA epimerase, mitochondrial

**Table 5.** Top 20 most abundant unigenes in *A. pegreffii* L4.

No.	ID	Name	Description
1	TBIU007721	PI-3	Major pepsin inhibitor 3
2	TBIU033076	col-145	Putative cuticle collagen 145
3	TBIU014455	col-34	Cuticle collagen 34
4	TBIU001741	col-34	Cuticle collagen 34
5	TBIU018580	ART2	Putative uncharacterized protein ART2
6	TBIU006603	COIII	Cytochrome c oxidase subunit 3
7	TBIU020343	col-138	Protein COL-138
8	TBIU020344	Bm1_54705	Nematode cuticle collagen N-terminal domain containing protein
9	TBIU013810	COI	Cytochrome c oxidase subunit 1
10	TBIU000598	col-40	Cuticle collagen 40
11	TBIU008478	COII	Cytochrome c oxidase subunit 2
12	TBIU003870	Y51F10.7	Protein Y51F10.7
13	TBIU015232	IXCI_RHIMP	Chymotrypsin-elastase inhibitor ixodidin
14	TBIU017350	ZK970.7	Protein ZK970.7
15	TBIU001757	Bm1_23600	hypothetical protein
16	TBIU015233	IXCI_RHIMP	Chymotrypsin-elastase inhibitor ixodidin
17	TBIU006107	col-182	Protein COL-182
18	TBIU029263	CRVP	Venom allergen 3
19	TBIU004632	col-34	Cuticle collagen 34
20	TBIU030958	LGALS8	Galectin-8

Different gene expression profiles between APL3 and APL4 transcriptomes were investigated by DEG analyses. In total, 1,923 genes were up-regulated either in APL3 or APL4. Among them, 614 were up-regulated in APL3 while 1,309 were up-regulated in APL4 (data not shown). The top 20 up-regulated genes either in APL3 or APL4 were listed in Tables 6 and 7.

**Table 6.** Top 20 most differentially expressed genes in *A. pegreffi* L3.

No.	Name	Description	Log FC	*P*-value	*Q*-value
1	ergic2	Endoplasmic reticulum-Golgi intermediate compartment protein 2	16.8	0.0001179	0.247384
2	mgat4b	Alpha-1,3-mannosyl-glycoprotein 4-beta-N-acetylglucosaminyltransferase B	16.7	0.0001274	0.247384
3	Acin1	Apoptotic chromatin condensation inducer in the nucleus	16.3	0.0002229	0.255429
4	SFXN2	Sideroflexin-2	16.1	0.0003069	0.265269
5	ANKHD1	Ankyrin repeat and KH domain-containing protein 1	16.1	0.0002901	0.265269
6	RALGAPA1	Ral GTPase-activating protein subunit alpha-1	15.9	0.0003816	0.278843
7	SI	Sucrase-isomaltase, intestinal	15.9	0.0004035	0.278843
8	B0361.11	Putative transporter B0361.11	15.9	0.0004042	0.278843
9	lact-2	Beta-lactamase domain-containing protein 2	15.8	0.0004392	0.291452
10	sptl-2	Serine palmitoyltransferase 2	15.8	0.0004306	0.291452
11	hyd	E3 ubiquitin-protein ligase hyd	15.7	0.0005016	0.292532
12	Macf1	Microtubule-actin cross-linking factor 1	15.7	0.0005216	0.292532
13	ZK370.4	Uncharacterized NTE family protein ZK370.4	15.7	0.000554	0.292532
14	LOAG_00120	Hypothetical protein	15.7	0.0005268	0.292532
15	FZD10	Frizzled-10	15.6	0.0005786	0.292532
16	cdgs-1	Phosphatidate cytidylyltransferase	15.6	0.0005679	0.292532
17	Pcsk1	Neuroendocrine convertase 1	15.6	0.000601	0.292532
18	Bm1_49625	Hypothetical protein	15.6	0.0006287	0.292532
19	GLSN_MEDSA	Glutamate synthase [NADH], amyloplastic	15.6	0.0006022	0.292532
20	Moe	Moesin/ezrin/radixin homolog 1	15.6	0.0005762	0.292532

**Table 7.** Top 20 most differentially expressed genes in *A. pegreffi* L4.

No	Name	Description	Log FC	p-value	q-value
1	F09E10.7	Protein F09E10.7	20.7	0.0000115	0.171197
2	ATP8B4	Probable phospholipid-transporting ATPase IM	18.7	0.0000172	0.171197
3	pept-1	Peptide transporter family 1	18.5	0.0000214	0.171197
4	vps-11	Vacuolar protein sorting-associated protein 11 homolog	18.5	0.0000214	0.171197
5	col-34	Cuticle collagen 34	18.4	0.0000229	0.171197
6	EGF1	Fibropellin-1	17.5	0.000063	0.206261
7	CLINT1	Clathrin interactor 1	17.4	0.0000751	0.218706
8	let-805	Protein LET-805	17.2	0.0000989	0.247384
9	Bm1_33530	Hypothetical protein	17	0.0001257	0.247384
10	VIT	Vitrin	17	0.0001194	0.247384
11	col-40	Cuticle collagen 40	16.9	0.0001524	0.255429
12	VIT	Vitrin	16.7	0.0002081	0.255429
13	unc-104	Kinesin-like protein unc-104	16.7	0.0001838	0.255429
14	SAM-MT	Probable fatty acid methyltransferase	16.7	0.000202	0.255429
15	sulp-3	Sulfate permease family protein 3	16.6	0.0002432	0.258155
16	CLINT1	Clathrin interactor 1	16.6	0.0002241	0.255429
17	Oplah	5-oxoprolinase	16.5	0.0002634	0.265269
18	VIT	Vitrin	16.4	0.0003171	0.265269
19	LOAG_09410	Hypothetical protein	16.4	0.0003238	0.265269
20	ZAN	Zonadhesin	16.4	0.0003204	0.265269

### GO enrichment analyses of DEGs in *A. pegreffii* L3 and L4 transcriptomes

**Figure 1:**
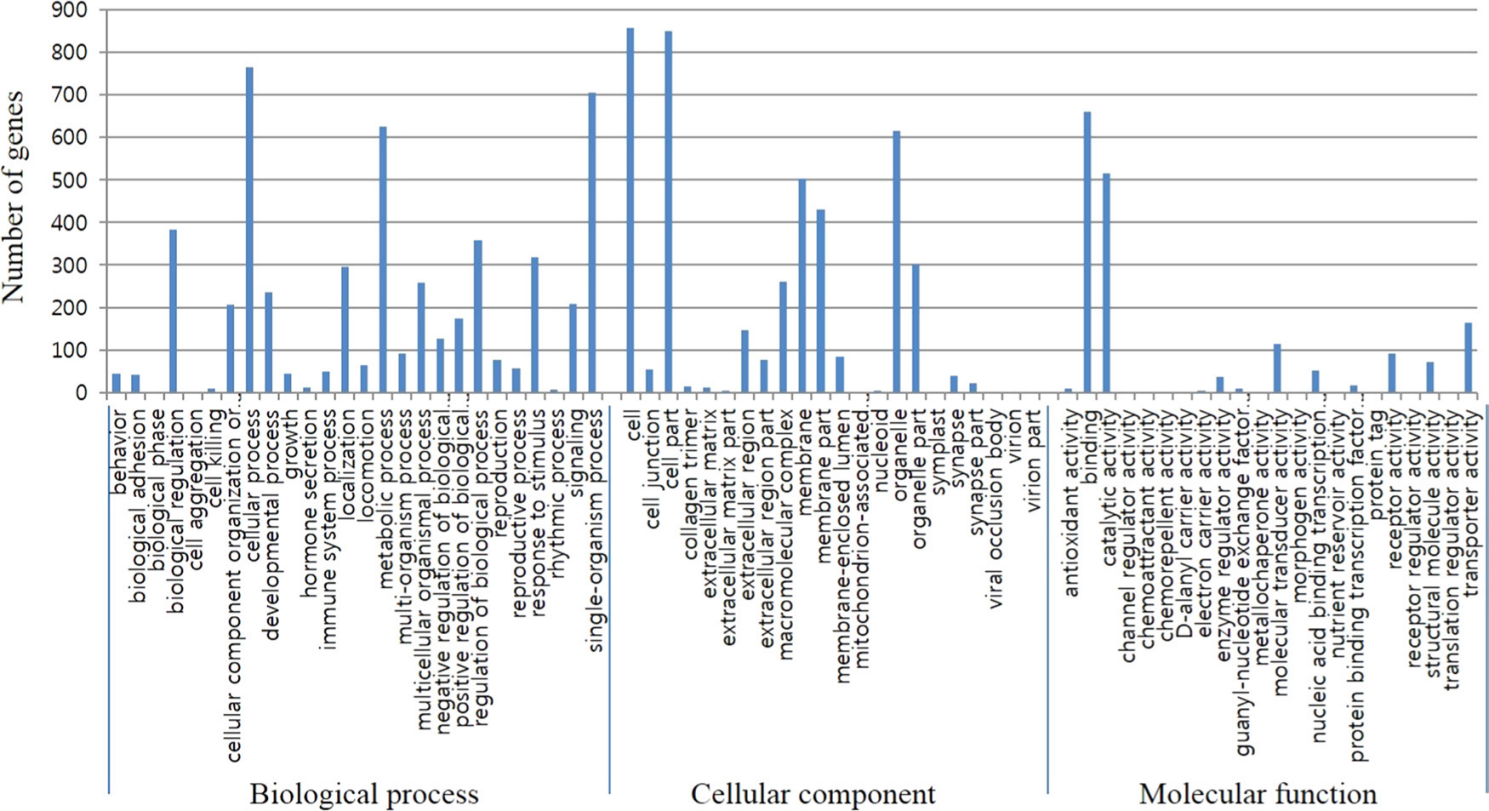
GO analysis of DEGs in *A. pegreffii* transcriptomes (*A. pegreffii* L3 vs *A. pegreffii* L4).

DEGs were subjected to GO analyses. In total 1,184 DEGs were mapped to either of three main categories (cellular component, biological process and molecular functions). GO analysis of the DEG revealed that 14 cellular component, 41 biological process, and 10 molecular function categories (*P* < 0.001) (data not shown). Cell, cellular process, and binding were the subcategories to which the largest number of genes belongs to each three categories (Fig. 1).

### Real-time PCR validation

**Figure 2:**
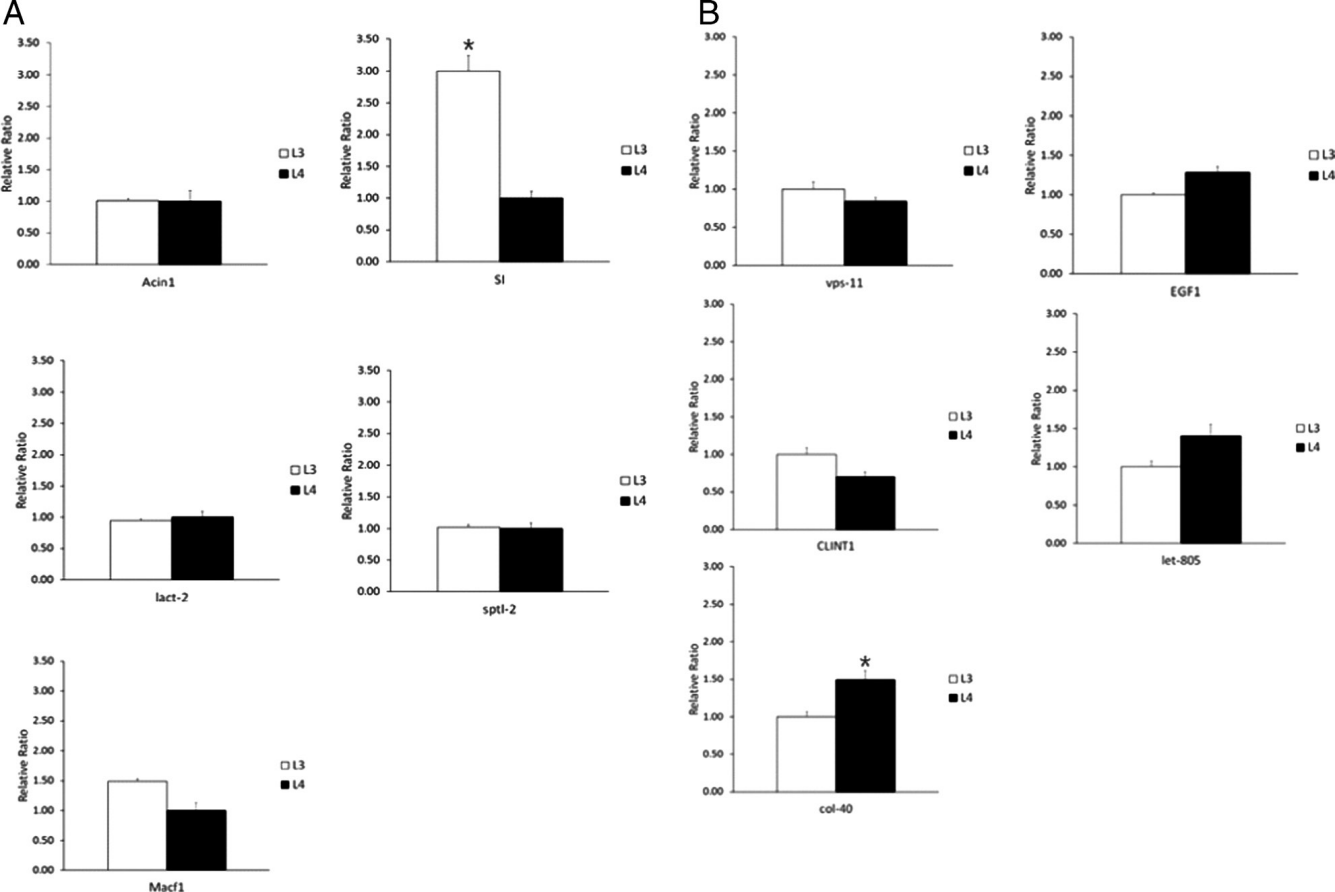

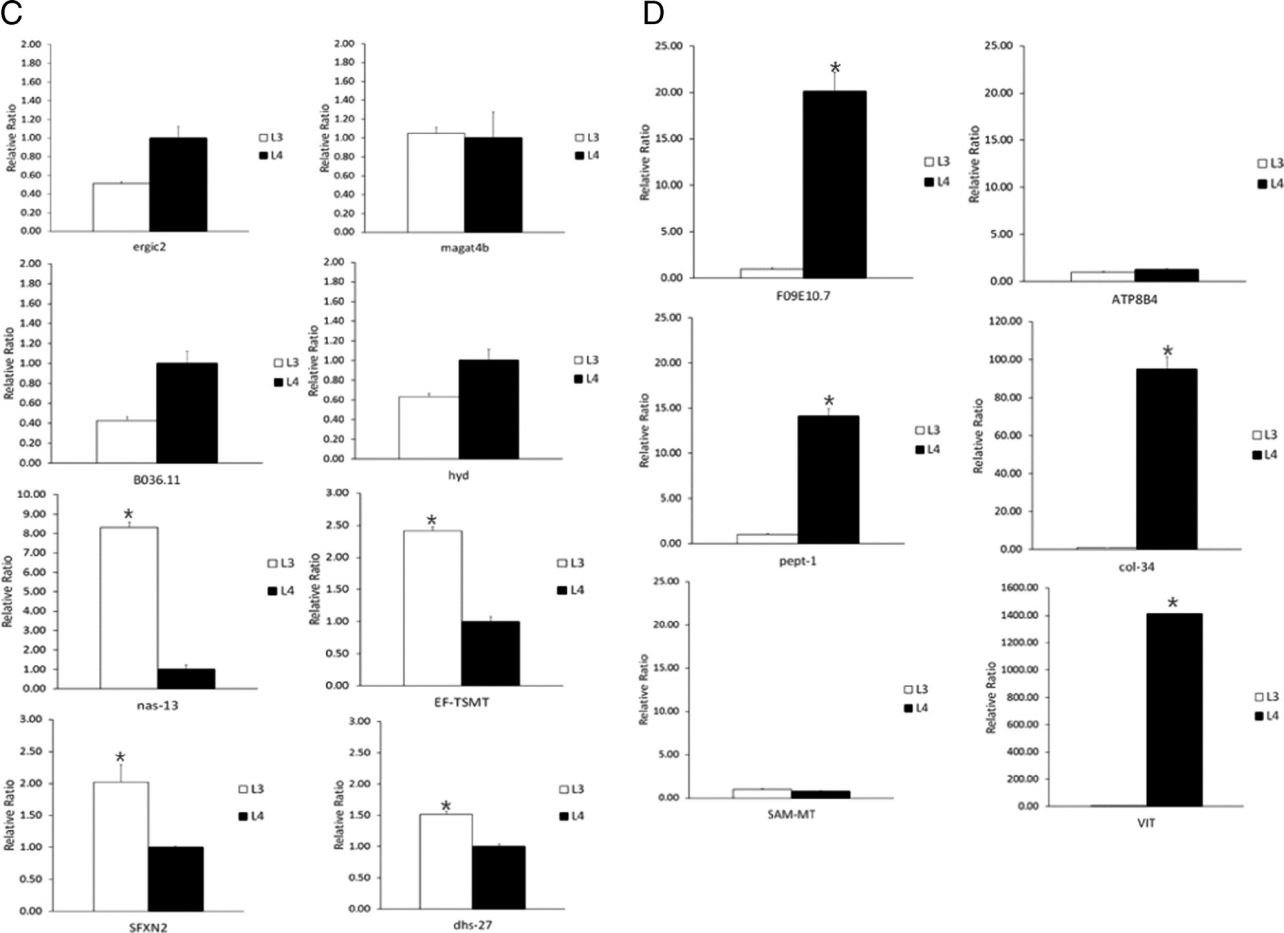
The expression level of selected DEGs either in APL3 or in APL4 by real-time PCR. A: the real-time PCR results of five genes with significantly higher expression level in APL3 compared to APL4; B: the real-time PCR results of five genes with significantly higher expression level in APL4 compared to APL3; C and D are 14 additional genes selected to compare the expression levels of ASL3 and APL4 (C: eight gene for APL3 compared to APL4, D: six gene for APL4 compared to APL3). GAPDH genes were used as the reference genes. The results are given as mean of samples (*n* = 3) and expressed as fold change in mRNA expression. *P < 0.05.

The mRNA expression level of the DEGs either in APL3 or APL4 were validated by real-time PCR. For APL3, the top 5 DEGs (Acin1, SI, lact-2, sptl-2, Macf1) were analyzed by real-time PCR, and only one gene (SI) was validated as significantly highly expressed in APL3 (*P* < 0.05; Fig. 2A). We additionally selected more DEGs for validation and four genes (nas-13, EF-TSMT, SFXN2, dhs-27) were highly expressed in APL3 (*P* < 0.05; Fig. 2C). For APL4, top 5 DEGs (vps-11, EGF1, CLINT1, let-805, col-40) were analyzed and only one gene (col-40) was validated as highly expressed (*P* < 0.05; Fig. 2B). Additionally selected DEGs were also analyzed and four genes (F09E10.7, pept-1, col-34, VIT) were highly expressed in APL4 (*P* < 0.05; Fig. 2D).

### Identification of putative allergens

Comparative analyses of *A. pegreffii* transcriptomes with the sequence data in the AllergenOnline database revealed that 42 transcripts corresponding to 17 different putative allergens and 14 transcripts to 8 previously reported *Anisakis* antigens (Tables S1 and S2 in https://doi.org/10.6084/m9.figshare.12090543). DEG analysis for the putative allergen sequences revealed that most of them were excluded in top 20 most DEGs lists of either APL3 or APL4 based on log FC value, except for 1 transcript (TBIU002596) corresponding to Ani s 11-like protein 2 precursor (Table S2 in https://doi.org/10.6084/m9.figshare.12090543).

## Discussion

*A. simplex* complex consist of three morphospecies: *A. simplex* (s.s.), *A. pegreffii* and *A. berlandi* (formerly named as *A. simplex* C) (Mattiucci et al., 2018), with different genetic structure, biology and geographical distribution. Of these three closely related species, *A. simplex* (s.s.) and *A. pegreffii* are known to sympatrically occur in several areas such as the Iberian Atlantic coast and Japanese Sea waters, where the putative hybrids have been reported as both of larvae and adults (Abollo et al., 2003; Umehara et al., 2006; Farjallah et al., 2008; Cavallero et al., 2014). Given that these two species frequently occur in commercially important marine fish species, and humans can become infected by eating raw or undercooked fish and cephalopod harboring these larvae, it is necessary to investigate their pathological potentials of both species. Several in vitro and in vivo studies demonstrated that both of these two species are invasive to cause harmful effects to humans (Suzuki et al., 2010; Jeon and Kim, 2015; Lee et al., 2017). Their pathologic potentials at transcriptomic levels were also recently compared (Cavallero et al., 2018) and partially differentially upregulated repertoires of transcripts were found in each species, but their detailed pathological meanings still await further study.

*A. simplex* has been known as the major cause of human infection, and therefore many publications exist regarding their pathological process. For example, the existence of several proteases was confirmed by either their biochemical activities or the sequences (Sakanari et al., 1989; Sakanari and McKerrow, 1990; Hotez et al., 1994; Quiazon et al., 2013). Currently several *A. simplex* transcriptome studies are available (Cavallero et al., 2018; Kim et al., 2018; Llorens et al., 2018), which made the systematic approach possible. However, *A. pegreffii*, a congeneric species of genus *Anisakis* is less investigated in terms of their genetic and biological characteristics; there is no reference genome available, thereby we conducted de novo transcriptome assembly with L3 and in vitro-induced of L4 of *A. pegreffii*, and compared their characteristics in this study.

Mitochondrial enzymes-related (COI, COII, COIII) and polyubiquitin-related genes (UNN, UBIQP_XENLA) were highly expressed in L3, while collagen-related genes (col-34, -145, 138, Bm1_54705) were highly expressed in L4. Mitochondrial cytochrome c oxidase is the key enzyme of aerobic cell respiration in all eukaryotes and many prokaryotes, and different level of this enzyme at different developmental stages of a parasitic nematode *Ascaris suum,* having the highest activity in L2 (the second stage larvae) and the lowest activity in adult stage, was reported (Takamiya et al., 1996). Adult anisakid nematodes are known to inhabit gastrointestinal tracts of marine vertebrates (Mattiucci et al., 2018), which probably have a low or fluctuating oxygen tension. Therefore, relatively lower activity of cytochrome oxidase would be expected with the development of anisakid nematodes larvae. But, the atmospheric oxygen concentrations of the hosts’ microhabitats for anisakid nematodes are currently unknown and should be investigated to make clear discussion.

Collagens are ubiquitous structural proteins constituting the cuticle, a multi-layered flexible exoskeleton for protecting nematodes from adverse environmental conditions (Page, 2013). A new cuticle is synthesized for each developmental stage of nematodes, with many enzymes being involved in this process. Large families of the cuticle collagen genes are known in *C. elegans*, and also in many parasitic nematodes (Page, 2013 and the references therein). In our study, collagen-related genes were abundantly transcribed in L4, suggesting new cuticles are actively synthesized.

Differential metabolic profiles among different developmental stages of several parasitic nematodes were shown by transcriptome analysis. A filarial nematode *Brugia malayi* showed different transcriptome repertoires of the overrepresented protease activity in L3 and the overrepresented structural component in L4 (Choi et al., 2011). A strongylid nematode *Haemonchus contortus* also showed the genes associated with oxygen transport and heme binding were up-regulated in L3, and the genes associated with body morphogenesis and various metabolic process were up-regulated in L4 (Laing et al., 2013). Parasitic nematodes should adapt themselves to different environments with differing food sources and energy requirements, and this is reflected in differential gene expression in different life cycle stages. Tyagi et al. (2015) recently reconstructed and compared metabolic pathways of 23 organisms including 13 nematode species, using their deduced proteomes. They observed that the metabolic potentials are in general concomitant with the phylogenetic and/or ecological similarity of the organisms investigated. Anisakid nematodes have complex life cycles involving aquatic invertebrates and vertebrates as intermediate or paratenic/transport hosts, and aquatic vertebrates as definitive hosts, which means they have to adapt to various environmental conditions and are equipped with diverse molecular repertoires to react with these dynamic circumstances from the hosts. Several transcriptomic studies of *Anisakis* species are currently available (Cavallero et al., 2018; Kim et al., 2018; Llorens et al., 2018), but information on transcriptomic changes during the life cycle are still scarce. In this study, we selected the highly ranked DEG either in L3 or in L4 and validated them by real-time PCR. Of 13 highly ranked differentially transcribed genes in L3, five genes (SI, nas-13, EF-TSMT, SFXN2, dhs-27) were validated by real-time PCR. For L4, five genes (col-40, F09E10.7, pept-1, col-34, VIT) were validated by real-time PCR from 11 highly ranked differentially transcribed genes. Some metabolic enzymes-related genes (e.g. SI, dhs-27) were more highly transcribed in L3 than L4, while some (e.g. PEPT-1) were more highly transcribed in L4 in this study. Many of these transcripts have orthologues in other species, which are so far uncharacterized and therefore await further study.

Allergic reactions are one of the clinical symptoms caused by *Anisakis* nematodes infection in humans (Nieuwenhuizen and Lopata, 2013; Baird et al., 2016). Currently, 15 allergens in total have been described from *A. simplex* (s.s.) according to the WHO/IUIS Allergen Nomenclature Database (Pomés et al., 2018), but the exact number and characteristics of potential allergen molecules in anisakid nematodes are yet to be defined. Recently Baird et al. (2016) and Llorens et al. (2018) analyzed allergens from larval *Anisakis* transcriptomes and described a number of putative allergens including previously reported *Anisakis* allergens. In this study, we listed 56 transcripts matching to consensus sequences from AllergenOnline database (e-value cut off: <1e-05, identity matching cut off: >70%). Of them, 42 transcripts were matched with those of allergens from other organisms. This might be related with cross-reactivity of the sera from *Anisakis*-induced allergic patients to allergens from other sources, as described by Llorens et al. (2018). 16 transcripts from *A. pegreffii* transcriptomes in this study were matched with novel *A. simplex* (s.s.) allergens, suggesting some of them commonly exist in *Anisakis* species. DEG analysis for these putative allergen sequences revealed that there were no considerable differences in log FC value between APL3 and APL4, except for one transcript corresponding to Ani s 11-like protein 2 precursor gene, which needs further validation. Different expression of allergen profiles depending on the life cycle stages of anisakid nematodes are yet to be reported.

While 15 allergens (Ani s 1 to Ani s 14 and Ani s troponin C) have been described from anisakid nematodes (Pomés et al., 2018), 8 *Anisakis* allergens including 2 unassigned ones were found from the AllergenOnline Database in the *A. pegreffii* transcriptomes in this study. Several explanations can be provided for this result; some of these *Anisakis* allergens might be unregistered in the database when browsed. Otherwise, some of the transcriptome profiles in our study might have been inevitably missed out because we cut a small portion of caudal end of each individual *A. pegreffii* larvae for molecular identification and conducted transcriptome analysis with the rest of them. Cavallero et al. (2018) recently conducted transcriptome analysis with whole *A. simplex* (s.s.) and APL3, and specifically focused their pharyngeal tissue transcriptome. Our methodological approach might have affected our transcriptome dataset profiles, consequently underestimated the total number of allergens in our data set.

There are several classes of proteases in nematodes (e.g. cysteine, serine, aspartic, and metalloproteases), with a variety of functions, and some of them are thought to be well conserved across the nematode species such as molting, embryogenesis or cellular survival. On the other hand, proteases of parasitic nematodes are also involved in diverse adaptive functions including host tissue penetration, larval migration, immune evasion, host tissue digestion, and so forth (Britton, 2013 and the references therein). Different parasitic nematode species and different developmental stages in a certain nematode species may have different repertoire of proteases for adapting themselves to a specific niche, and comparative analyses of these differences would be essential to understand their pathogenesis and develop potential control strategies. For example, transcriptome analysis of a parasitic nematode *Angiostrongylus cantonensis* revealed that the infective third stage larvae highly expressed metallo-, aspartic-, and cysteine protease, while the fifth stage larvae highly expressed cysteine-, aspartic-, and serine proteases (Chang et al., 2014), suggesting these proteases have different roles in different developmental stages of parasites. Moreover, transcript profiles of aspartic protease genes (e.g., Sc-ASP110, 113, 155) in an entomopathogenic nematode *Steinernema carpocapsae* were characterized and different expression profiles of each transcript were found in the different developmental stages (Balasubramanian, Nascimento, Ferreira, Martinez, and Simões, 2012; Balasubramanian, Toubarro, Nascimento, Ferreira, and Simões, 2012; Balasubramanian and Simões, 2013). Of genes investigated in this study, many cysteine protease (Atg4c, atg4a, clp-1 G1), metalloprotease (TM104_CAEBR, ccpp-6, dpy-31), aspartic protease (asp-6 G1, asp-6 G2), hyaluronidase (HYAL1), enolase (enol-1)-related genes were found to be highly transcribed in APL3 by real-time PCR, while clp-1 G2 was validated to be highly transcribed in APL4 (Fig. 2). The third stage larvae of parasitic nematodes should successfully establish infection in the final hosts, and have many strategies to evade host defense mechanisms. The proteases secreted in third stage larvae of parasitic nematodes are therefore thought to work as one of the strategies to evade host defense mechanisms. Several proteases of *A. simplex* have been reported (Sakanari et al., 1989, Sakanari and McKerrow, 1990; Hotez et al., 1994). Lee et al. (2017) reported strong activities of matrix metalloproteinases and serine proteases in excretory/secretory product of *A. pegreffii* infective third stage larvae, which is thought to support our findings. In particular, Cavallero et al. (2018) recently analyzed pharyngeal tissue transcriptomes of 2 sibling *Anisakis* species (*A. simplex* (s.s.) and *A. pegreffii*) and found upregulated transcripts involving pathogenicity (e.g. proteolysis, anesthesia, hemostasis inhibitors, anticoagulant, virulence factors, and immunomodulatory peptides) in the pharyngeal transcriptomes, when compared with the corresponding whole larvae transcriptomes. They suggested that these DEGs are potentially involved in host tissue invasion and pathogenesis. In this study, we also found different DEG repertoires between APL3 and APL4, mainly proteolytic enzymes and cuticle synthesis as mentioned above.

While we report comparative transcriptomic analyses of different developmental stages of *A. pegreffii* in this study, there are still much gaps in our knowledge of many DEGs genes either in L3 or in L4. Currently, the draft genome of *A. pegreffii* is not available. The draft whole genome of a sibling species *A. simplex* was recently uploaded (https://www.ncbi.nlm.nih.gov/assembly/GCA_900617985.1), yet to be completely annotated. Thus, the number of total orthologue genes in *A. pegreffii* transcriptome and DEGs either in L3 or in L4 might be underestimated. More precise information on the predicted gene functions of *A. pegreffii* transcriptomes will be obtained when the reference genome sequences are available and in vitro experiments are conducted.
